# Supplementary figures and images for: Control of SARS-CoV-2 infection after Spike DNA or Spike DNA+Protein co-immunization in rhesus macaques
Source: PLoS Pathog. 2021 Sep 22;17(9):e1009701. doi: 10.1371/journal.ppat.1009701 (PMC8489704; doi:10.1371/journal.ppat.1009701)

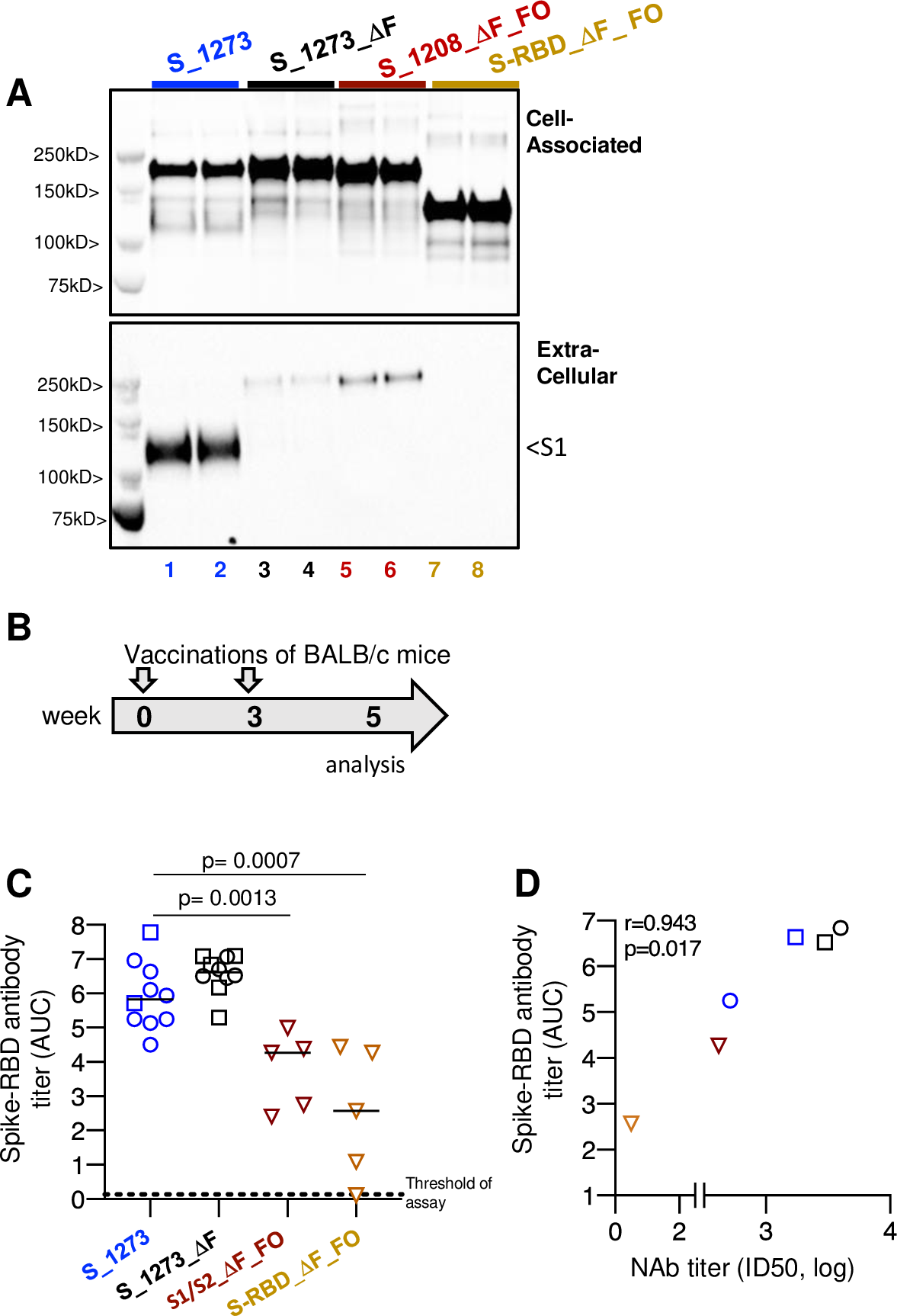

Supplement: S1 Fig — (A) Western blot analysis of transfected HEK293 cells detects the Spike proteins associated with the cell (top panel) and supernatant (bottom panel) using an S1-Spike specific antibody. Expression of S-1273 DNA resulted in the production and secretion of the cleaved S1 product while full-length Spike was found in the cell-associated fraction (lanes 1–2). All Spike proteins with the ΔF mutation (lanes 3–4 and lanes 5–6, respectively), including the S-RBD (lanes 7–8), remain mostly in the cell-associated fraction. Duplicate transfections are shown. (B-D) Mice were vaccinated twice (week 0, 3) with the indicated DNAs. (C) Anti-Spike-RBD antibody responses were measured by ELISA and shown as AUC titer (log) established by model fit approach. Open circle and square symbols for the S_1273 and the S_1273_ΔF groups denote two independent studies which showed similar anti-Spike-RBD antibody levels, presented here as pooled data. Median values are indicated. P values are from non-parametric ANOVA (Kruskal-Wallis test). (D) Correlation of Spike-RBD ELISA titers (AUC) and reciprocal pseudotype NAb ID50 titer (log). (TIF) [file ppat.1009701.s001.tif]

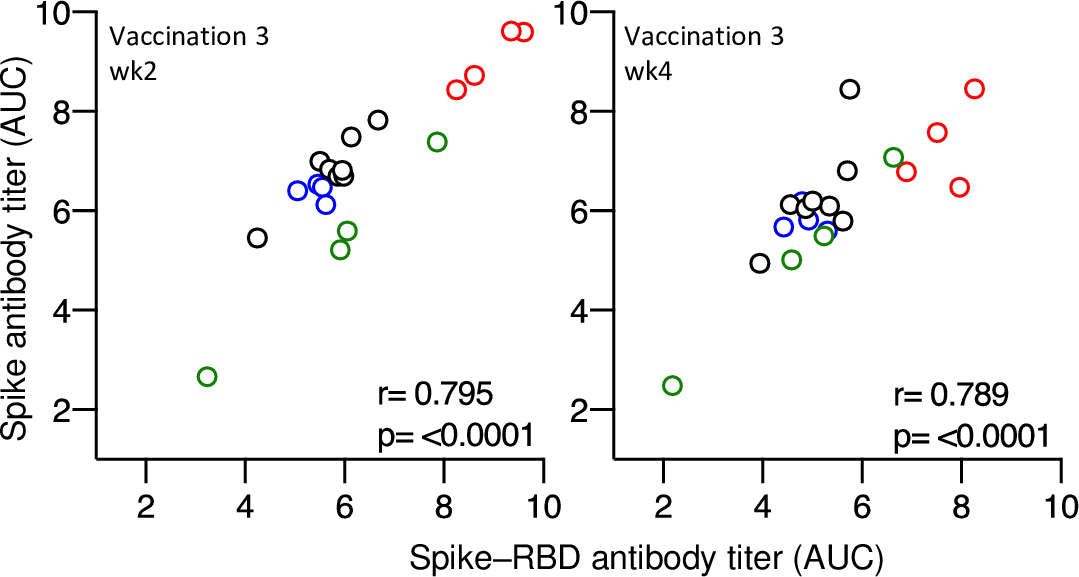

Supplement: S2 Fig — ELISA measuring antibodies against S-RBD and trimeric Spike proteins in plasma collected at 2 and 4 weeks after the 3rd vaccination showing excellent correlations among the assays. (TIF) [file ppat.1009701.s002.tif]

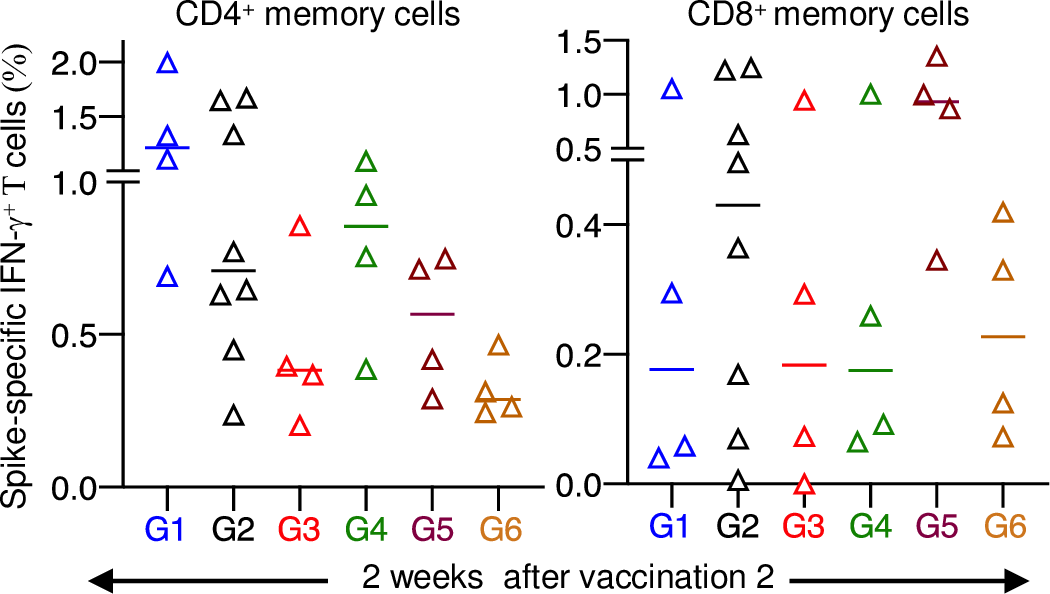

Supplement: S3 Fig — Spike-specific IFN-γ+ memory T cell responses, measured 2 weeks after the 2nd vaccination of all groups are shown as % of memory CD4+ (left panel) and as % of memory CD8+ (right panel) T cell subset. The data from G4 and G5 are also shown in Fig 2D. Median values are indicated. (TIF) [file ppat.1009701.s003.tif]

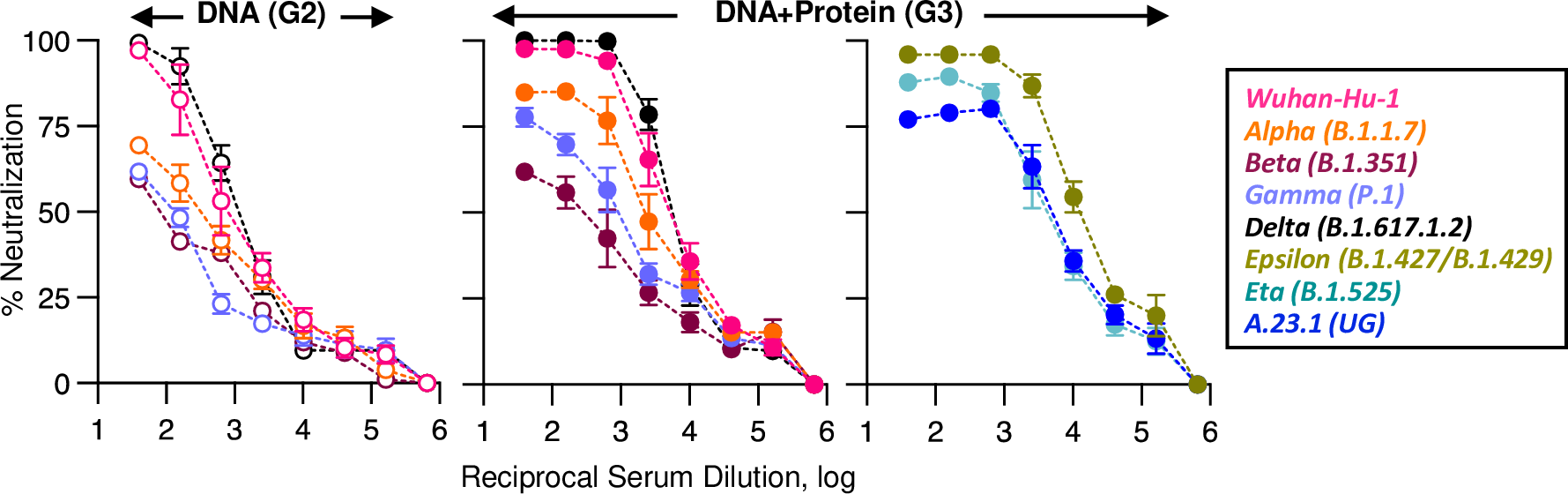

Supplement: S4 Fig — The % neutralization of the DNA-only (G2) and the DNA+Protein (G3) vaccinated macaques against a panel of Spike variants are shown. Neutralization is calculated for each assay and plotted over the serial reciprocal serum dilutions. Mean and SEM are shown. (TIF) [file ppat.1009701.s004.tif]
