# Supplementary material for: Control of SARS-CoV-2 infection after Spike DNA or Spike DNA+Protein co-immunization in rhesus macaques
Source: PLoS Pathog. 2021 Sep 22;17(9):e1009701. doi: 10.1371/journal.ppat.1009701 (PMC8489704; doi:10.1371/journal.ppat.1009701)
Supplement: Attachment — Submitted filename: Reviewer response08252021.docx [file ppat.1009701.s005.docx]

**POINT-BY-POINT REPLY TO REVIEWERS’ COMMENTS:**

Reviewer Comments (if any, and for reference):

Reviewer's Responses to Questions

***Part I - Summary*** *Please use this section to discuss strengths/weaknesses of study, novelty/significance, general execution and scholarship.*

*Reviewer #1: Rosati et al. report a preclinical trials testing vaccines aimed at protecting from SARS-CoV-2 infection. They investigated two platforms, DNA alone and DNA+protein, that focused on the Spike protein included in different configurations as CoV-2 targeted antigen. The results indicated that while all vaccines provided protection for lower airways infection, the Spike DNA+ protein platform provided the best level of binding and neutralizing antibody and the strongest control of the infection. The vaccination follow up is carefully designed and the immunization evaluation is reported in detail. This study would benefit by is the evaluation of the immunity induced in macaques against the delta variant that is at present the most significant SARS-CoV-2 circulating variant.*

OUR RESPONSE: We have added data with the Delta variant in the revised manuscript (Figures 3B, 3C, S4 Fig). We modified the abstract (lane 40-42) and result section (lanes 220-270), accordingly.

*Reviewer #2: Rosati M et al, report immunogenicity and protective efficacy of a DNA based SARS-CoV-2 targeted vaccine regimen expressing different pre-fusion stabilized Spike immunogens used via the I.M route of vaccination followed by electroporation in the rhesus macaque model. The authors build on their vast experience with pre-clinical studies with the DNA vaccine platform tested previously with HIV vaccine studies in the macaque model and specifically highlight the merits of combining DNA vaccines with protein+adjuvant (GLA-SE) co-delivered at the same anatomical site.
Overall, the article is very well written, the data is quite straightforward in the context of SARS-CoV-2 vaccine pre-clinical studies now extensively studied and documented. As a reviewer, I would argue that a technique with electroporation for mass vaccinations will potentially have less appeal in an ongoing pandemic only further complicated with a co-delivery of a protein vaccination with an adjuvant. Furthermore, the fact that mRNA vaccines have demonstrated the best-in-class immune responses (acute, while durability is still being investigated) and protective efficacies, the clinical translation of DNA vaccines in general and a co-delivery of DNA+Protein based immunization for SARS-CoV-2 looks challenging or less appealing at this stage of the pandemic. Having said that, like “all hands-on deck” when it comes to helping out with the pandemic, I submit that all vaccine platform approaches and data generated in pre-clinical studies, specifically with non-human primates (NHPs) are extremely useful in iterative design of future studies with other vaccine platforms. Based on this argument and the fact that the authors have done a really nice job in writing this article, I recommend publication of this work pending clarification of a couple of comments below.*

OUR RESPONSE: We thank the reviewer for the positive response. Indeed, when this work started in March 2020, the success of the mRNA was not predictable. On the other hand, it turns out that SARS-CoV-2 Spike is an excellent immunogen to test the potency of additional vaccine platforms or vaccine platform improvements with possible applications also different from COVID.

**Part II – Major Issues: Key Experiments Required for Acceptance**
*Please use this section to detail the key new experiments or modifications of existing experiments that should be absolutely required to validate study conclusions.

Generally, there should be no more than 3 such required experiments or major modifications for a "Major Revision" recommendation. If more than 3 experiments are necessary to validate the study conclusions, then you are encouraged to recommend "Reject".*

*Reviewer #1: Considering that at present the most significant circulating variant is the delta variant and that the authors have tested the vaccine induced immunity against multiple variants that are less significant, this study would benefit by reporting in figure 3B the evaluation of the vaccine-induced immunity against the variant B.1.617.2. Obviously, it is easy to make a DNA vaccine that is specific for this variant, but it remains interesting to know how the immunity induced by a vaccine including a Spike sequence based on those used in human vaccination behaves against B.1.617.2 SARS-CoV-2.*

OUR RESPONSE: We have added data with Delta variant in the revised manuscript (Figures 3B, 3C, S4 Fig). The new panel (Fig 3B) shows cross-binding ELISA assay using Alpha, Beta and Delta. Fig 3C shows neutralizing ability against Delta as well as several variants. We modified the abstract (lane 40-42) the result section (lanes 233; 253-255; 260-269) and Material and Method section (lane 721) accordingly.

*Reviewer #2: 1. What was the criteria used to select treatment groups to be included in the challenge analyses?
a. One biggest disappointment for me a reviewer is the exclusion of group 5 from challenge. Group 5 had the best outcome with CD8+ T cell responses in comparison with group 2 which had higher Ab responses. The idea that induction of CD8+ T cell responses could benefit in the context of waning Ab based immunity would have been very interesting to document in the context of protective efficacy. Please discuss potential benefits of such a finding in potential future studies with heterologous challenge with SARS-CoV-2 variants.*

OUR RESPONSE: We agree with the reviewer, and this was the actual plan of the study. We and several other groups became victim of using a mutant SARS-CoV-2 virus stock that was provided by BEI. This mutant virus grew perfectly in Vero cells, was infectious in Vero cells but was not infectious in macaques. This unfortunate fact became apparent only after the vaccinated animals and controls were necropsied and analyzed. Subsequently, all virus stocks were analyzed, and the mutation was verified. A different and verified to be pathogenic virus stock was used to infect the animals in the challenge part of the presented study (Groups 1-4 and controls).

We also agree that this concept should be tested in future studies, and we are working on further improving the DNA vaccine immunogen and delivery methods.

*2. The authors have done a good job in ensuring that Ab responses one week prior to challenge was correlated with protection outcome rather than peak values that highlights some sense of durability of immune responses. However, since most of the reporting is done in human trials for the peak 2 week time point after a vaccination regimen, I feel in Figure 4, it would be good to have a panel of plots highlighting potential correlates with 2 week Ab responses after the last vaccination for a side by side comparison with the durable Ab response correlate. This would highlight if there is differential waning of either binding, nAb or effector functions overall.*

OUR RESPONSE: We have added data from 2 weeks post the last vaccination which are shown in Figure 4C-4E and we removed these data from S5 Fig. in the revised manuscript. The data of the correlations with measurements 1 week before challenge are now shown in Fig4F-I. Comparison of the 2 data sets (2 and 4 weeks post last vaccination corresponding to 3 and 1 week before challenge) showed that the same conclusions could be drawn using either of the data sets.

*3. Do T cell responses either 2 weeks or 4 weeks after the challenge correlate with VLs in nasal swabs? Won’t it be good to also include that data since robust CD4+ T cell responses were observed ?*

OUR RESPONSE: We added the following sentence on lane 363:

“*In contrast, the pre-challenge vaccine-induced T cell responses did not correlate with virus control, likely due to the strong contribution of the humoral immune responses and the very short course of infection (few days) in this model.”*

*4. The authors discuss the potential advantages of a balanced humoral vs. cellular immunity afforded by DNA vaccines and here with DNA+Protein vaccines (lines 367-380). However, the critical observation is that the third vaccination could have really amplified these responses and in all fairness the emergency use approved mRNA vaccines use only 2 rounds of vaccinations. It remains to be seen whether the booster or the 3rd vaccination if approved will strongly amplify CD4+ and CD8+ T cell responses. So, at present, I would cautiously claim advantages of the DNA vaccine approach relative to mRNA vaccines in their ability to induce stronger cellular immunity.*

OUR RESPONSE: We have modified the text, lane 455:

*“It is possible that a 3^rd^ booster mRNA vaccination may alleviate this. Our overall experience with DNA vaccines has been that they provide superior cellular immunity in numbers and duration, and the hypothesis to test is that it may be of benefit for the establishment of long-term protective immunity.”*

*5. Most journals and recent articles use the WHO nomenclature for identifying variants and would recommend that the authors refer to the variants referred to in the text and in figures as Alpha, Beta, Gamma variants….*

OUR RESPONSE: We have added the new names in the revised manuscript

**Part III – Minor Issues: Editorial and Data Presentation Modifications**
*Please use this section for editorial suggestions as well as relatively minor modifications of existing data that would enhance clarity.*

*Reviewer #1: As the foldon is not present in the challenge virus, it may provide a tool to measure persistence of the immune response by evaluating antibodies against its sequence even after vial challenge. This might be an interesting piece of information for the vaccine platforms used in this study if the animals are still available and sufficient time has passed since the evaluation of protection against challenge.*

OUR RESPONSE: See also our response above to reviewer 2. We agree with the reviewer and this was the actual plan of the study. We and others became victim of using a mutant non-pathogenic SARS-CoV-2 virus stock provided by BEI. This mutant virus grew perfectly in Vero cells, was infectious in Vero cells but was not infectious in macaques. This unfortunate fact became apparent only after the vaccinated animals and controls were necropsied and analyzed. Subsequently, all virus stocks were analyzed and the mutation in some batches were verified. A different and verified to be pathogenic virus stock was used to infect the animals in the challenge part of the presented study (Groups 1-4 and controls).

*Reviewer #2: Minor comments
1. Please indicate the # of animals in each treatment group in the macaque study on line 131 and also in Figure 1 as it is very difficult to get a good idea on the animal number.*

OUR RESPONSE: We modified Figure 1A and added the info on line 131: ”4 animals per group, except group 2 with 8 animals”.

*2. Is titer computed as an AUC titer or endpoint? The authors are not consistent with terminology as the Y axis in Figure 1B says AUC and the results section on line 157 refer to endpoint titers followed by AUC titers on line 164. Please be consistent when detailing data.*

OUR RESPONSE:

We corrected the Figure S1 Fig legend and text lane 123-125 reporting AUC titers.

We clarified the text in the revised manuscript (lane 162-163 corresponding to lane 157 in the original manuscript): “…..~7-fold higher level after the last vaccination based on a linear antibody endpoint titer comparison.”

And we add a sentence in the M&M section (lane 535-536): “…Comparison of antibody levels were performed using linear endpoint titer values.”
